# Bronchogenic cyst at esophagogastric junction treated by laparoscopic full-thickness resection and hand-sewn closure: a case report

**DOI:** 10.1186/s40792-016-0168-z

**Published:** 2016-04-27

**Authors:** Akiko Tonouchi, Takahiro Kinoshita, Hideki Sunagawa, Takuya Hamakawa, Akio Kaito, Hidehito Shibasaki, Takeshi Kuwata, Yosuke Seki, Toshirou Nishida

**Affiliations:** Gastric Surgery Division, National Cancer Centre Hospital East, 6-5-1 Kashiwanoha, Kashiwa, Chiba 277-8577 Japan; Pathological Division, National Cancer Centre Hospital East, Kashiwa, Japan; Weight Loss and Metabolic Surgery Center, Yotsuya Medical Cube, Tokyo, Japan

**Keywords:** Bronchogenic cyst, Esophagogastric junction, Laparoscopic resection

## Abstract

**Background:**

We herein report a case of a bronchogenic cyst arising from the esophagogastric junction treated by laparoscopic full-thickness extirpation. The full-thickness defect was closed by hand sewing a T-shaped line over the gastroendoscope as a bougie to prevent postoperative deformity or stenosis. Partial fundoplication (Toupet fundoplication) was added to prevent reflux.

**Case presentation:**

A 32-year-old woman with a body mass index of 43 kg/m^2^ was admitted for treatment of a cyst-forming submucosal tumor (60 mm in diameter) on the anterior wall of the esophagogastric junction, which was detected during screening endoscopy before bariatric surgery. The tumor was an extraluminal growing type but exhibited severe erosion at the mucosal site. A cystic tumor such as a duplication cyst, bronchogenic cyst, or cyst-forming gastrointestinal stromal tumor was suspected, and the abovementioned surgery was carried out. The postoperative course was uneventful. The pathological findings revealed the tumor to be a benign bronchogenic cyst. Endoscopic examination 3 months postoperatively showed no deformity or stenosis, and the patient complained of no reflux symptoms.

**Conclusion:**

This procedure may be an efficient option for treatment of submucosal tumors on the esophagogastric junction to maintain function or avoid excessive surgery.

## Background

Bronchogenic cysts are one of several bronchopulmonary foregut malformations. The most common location is in the mediastinum. Bronchogenic cysts in the abdominal cavity are rare, but the esophagogastric junction (EGJ) is reportedly a relatively common site [1]. Surgical resection is usually recommended because of the risk of malignant transformation. Simple excision of the tumor is usually performed, but major surgical resection has also been reported [2]. Laparoscopic excision of intra-abdominal bronchogenic cysts located close to the EGJ was recently reported as a less invasive procedure. However, because of the complex anatomy around the EGJ and rarity of the disease, the standard resection method has not been established, and the optimal procedure should be chosen according to the individual patient’s situation. We herein report a case of a bronchogenic cyst arising from the EGJ in a severely obese woman. The cyst was successfully treated by laparoscopic full-thickness resection followed by careful hand-sewn closure and prevention of reflux.

## Case presentation

**Fig. 1:**
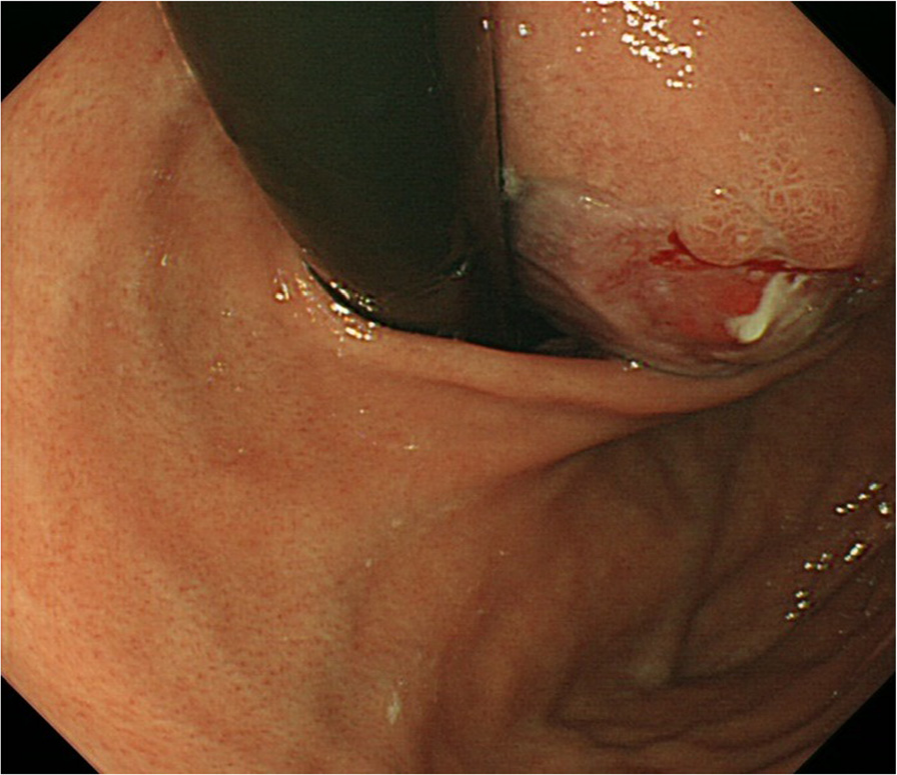
Upper endoscopy showed a 60-mm submucosal tumor with mucosal ulceration on the top. The tumor extended from the lower esophagus to the upper gastric corpus

**Fig. 2:**
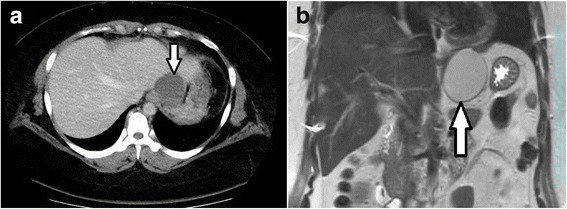
**a** Preoperative computed tomography showed the cystic tumor attached to the anterior wall of the esophagus and EGJ. **b** T2-weighted magnetic resonance imaging (*coronal view*) showed a high-intensity mass between the liver and stomach

A 32-year-old woman with a body mass index of 43 kg/m^2^ presented to the bariatric surgery center at another hospital. Sleeve gastrectomy was scheduled, but gastroendoscopy screening revealed a submucosal tumor (SMT) on the EGJ. She was referred to us for further evaluation. Endoscopy findings showed an SMT on the anterior wall of the EGJ with severe erosion on its mucosal surface. The epicenter was estimated to be 10 mm distal to the EGJ (Fig. 1). Abdominal computed tomography showed a low-density cystic lesion (60 mm in diameter), and magnetic resonance imaging demonstrated a homogenous cystic mass with iso-intensity on T1-weighted images, high intensity on T2-weighted images, and continuity with the intestinal wall (Fig. 2). Fine needle aspiration biopsy was not performed because of the cystic contents, but differential diagnoses included a cystic tumor such as a duplication cyst, bronchogenic cyst, or cyst-forming gastrointestinal stromal tumor.

**Fig. 3:**
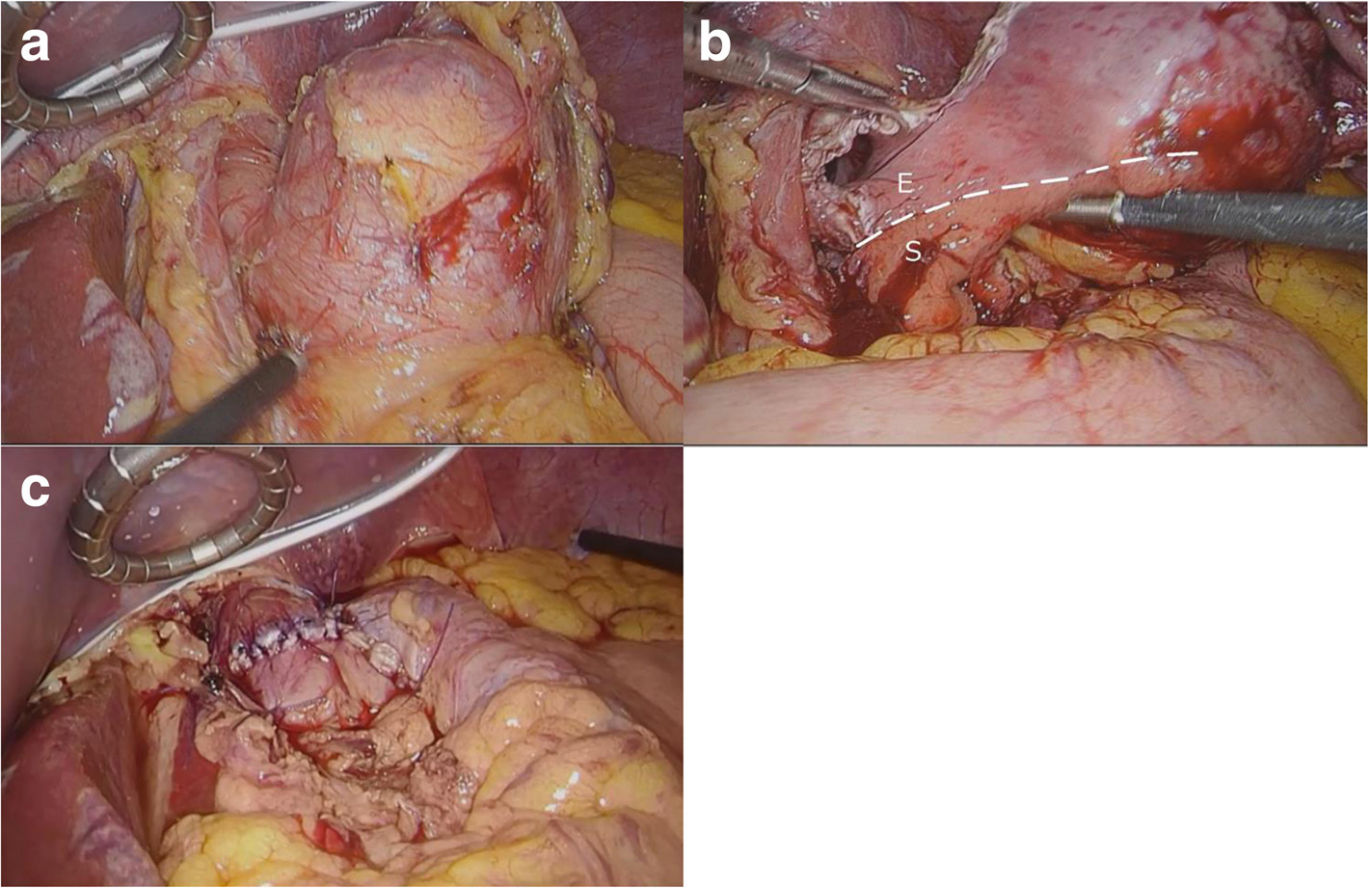
**a** The tumor was attached to the EGJ. **b** Full-thickness dissection was performed (*E* esophagus, *S* stomach). **c** The defect on the EGJ was closed by hand sewing. The esophageal side was closed vertically with interrupted sutures. The gastric side was closed horizontally by a running suture. Toupet fundoplication was added

**Fig. 4:**
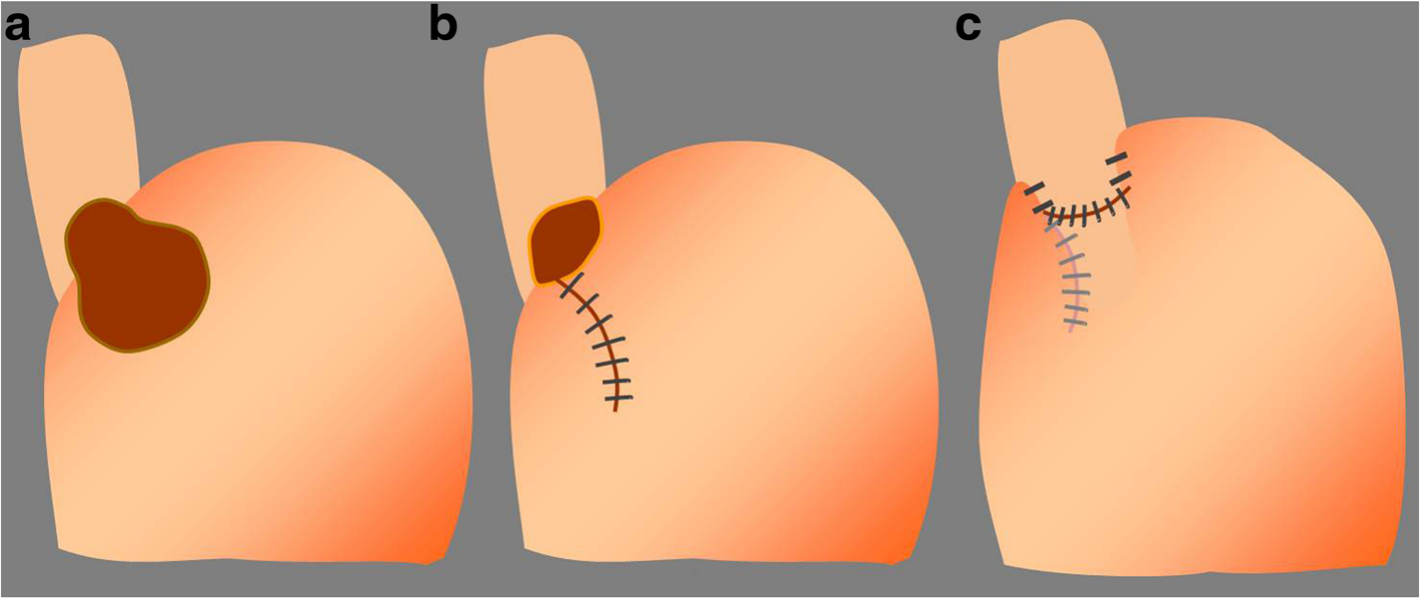
Schemas of the operative procedures. **a** After resection of the tumor, **b** the stomach defect was closed by a continuous suture, **c** the esophageal defect was closed by interrupted sutures, and Toupet fundoplication was added

**Fig. 5:**
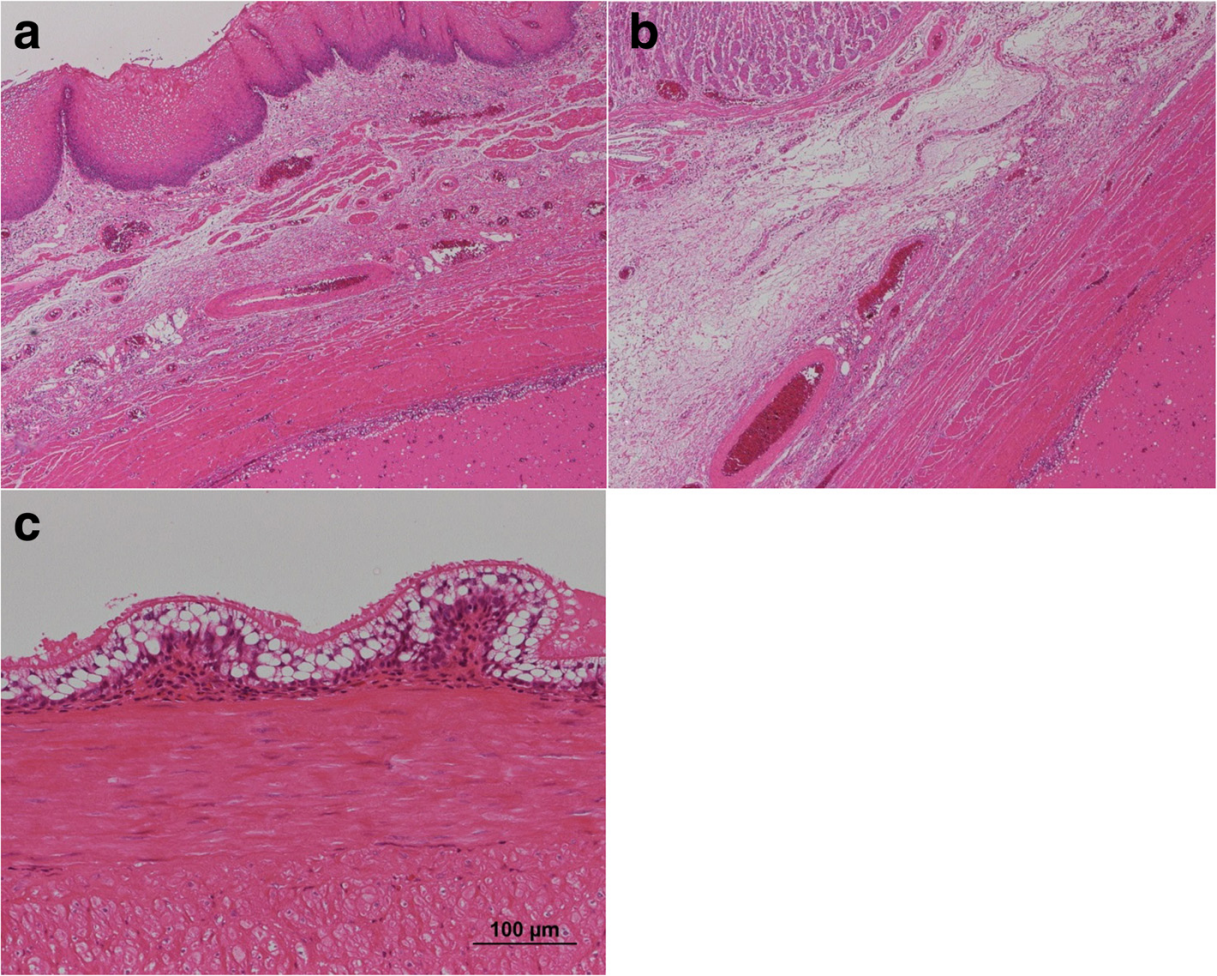
Pathological findings. **a**, **b** The surface of the tumor was covered with the mucosal layer of the esophagus and stomach in continuity with their muscular layer. **c** The intraluminal surface of the tumor was lined with columnar ciliated epithelium (hematoxylin and eosin staining)

Although the tumor was over 50 mm in diameter, after considering the low probability of malignant disease in this patient and our surgical team’s broad experience with laparoscopic surgery, we selected laparoscopic extirpation of this SMT. We considered that full-thickness resection, not enucleation, was required because of the presence of severe mucosal erosion. The patient was obese enough to be a bariatric surgery candidate; therefore, the operation was carried out with the cooperation of gastric surgeons and an expert bariatric surgeon. First, a 12-mm trocar for the scope was inserted at the umbilicus using an optical method; next, two 5-mm trocars were inserted in the bilateral subcostal region, two 12-mm trocars were inserted in the bilateral flank region, and another 5-mm trocar was inserted in the epigastric region for liver retraction. The lateral segment of the liver was retracted using Silicon Disc™ (Hakko, Nagano, Japan) and snake retractor to ensure adequate visibility around the EGJ. The lesser omentum was dissected to expose the abdominal esophagus. A smooth extraluminal tumor originating from the right side of the EGJ was seen. The base of the tumor was carefully exposed and excised circumferentially to its full thickness using the ultrasonically activated device (Fig. 3). The defect was relatively large, especially on the stomach side. For closure, the stomach wall site was first closed with a continuous suture in the direction of the long axis to adjust the rim length of the gastric and esophageal sites. The esophageal wall and stomach wall were then closed by interrupted sutures, finally resulting in a T shape (Fig. 4). The latter suturing was performed over the gastroendoscope as a bougie to prevent stricture. An air leakage test was performed to verify the tightness. Finally, Toupet fundoplication was added as an anti-reflux procedure. The resected specimen was removed from the enlarged umbilical port in an extraction bag. The operation time was 219 min. Gross examination of the extracted specimen showed a monolocular cystic mass covered with a fibrous capsule-like surface and containing brownish viscid fluid. Pathological examination showed that the surface of the cyst was covered with the mucosal layer of the esophagus and stomach in continuity with its muscular layer. The luminal surface was lined by columnar ciliated epithelium (Fig. 5). There were no malignant findings. The tumor was finally diagnosed as a bronchogenic cyst arising from the EGJ.

**Fig. 6:**
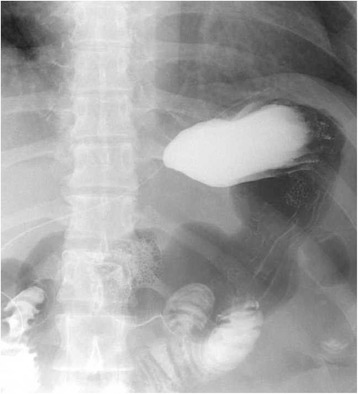
Postoperative esophagography using Gastrografin, which revealed no reflux in a head-down-tilted position

**Fig. 7:**
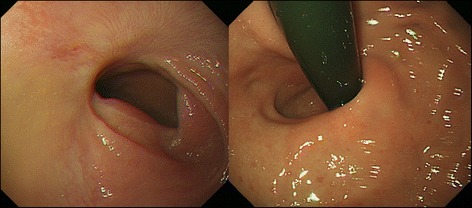
Endoscopic evaluation 3 months postoperatively showed no stenosis, deformation of the cardia, or reflux

The postoperative course was uneventful. Esophagography performed 3 days after the surgery using Gastrografin showed no leakage or stenosis; also, reflux to the esophagus was not observed, even in head-down-tilted position (Fig. 6). She was discharged on postoperative day 8. Gastroendoscopy examination showed no deformity or stenosis 3 months postoperatively, and the patient complained of no reflux symptoms (Fig. 7).

### Discussion

Bronchogenic cysts arise due to a lack of continuity between the tracheobronchial tract and the lung buds after abnormal germination and segregation. Until week 7 of gestation, when the pleural and peritoneal cavities are separated by the formation of the diaphragm, these cavities are formed intra-abdominally [1]. Most bronchogenic cysts are located in the mediastinum or intrapulmonary region; sub-diaphragmatic formation has been rarely described. Differential diagnoses are often extensive and include gastrointestinal stromal tumors, leiomyomas, lymphangiomas, and duplication cysts. Imaging examination shows a simple cystic spherical lesion but is not satisfactory as a definitive diagnostic modality [3, 4]. Some authors have reported that endoscopic ultrasound-guided fine needle aspiration cytology showing ciliated respiratory mucosa was helpful for the diagnosis [5], but preoperative diagnosis generally seems difficult. Most affected patients are asymptomatic, but some complain of epigastric pain, nausea, or vomiting. If an esophageal bronchogenic cyst is left untreated, complications such as infection, ulceration, intracystic bleeding, fistula formation, and malignant transformation may occur [5], particularly in adulthood; therefore, surgical treatment is recommended. Kirmani et al. [6] reviewed the treatment results for both symptomatic and asymptomatic bronchogenic cysts and found that 45 % of the asymptomatic patients subsequently developed symptoms; moreover, the operations for symptomatic cysts often became complicated.

Surgical excision of SMTs on the EGJ remains challenging because of the anatomical complexity and delicate function. Clinical decision-making may need to be employed with consideration of the individual patient. Wedge resection involving the EGJ using a stapler seems unsuitable because of postoperative deformation and stenosis. Proximal gastrectomy with lower esophageal resection will lead to remarkable decline in the patient’s quality of life. As alternatives, several novel techniques with which to minimize the extent of resection of the normal gastrointestinal tract wall were recently published, such as laparoscopic and endoscopic cooperative surgery or percutaneous endoscopic intragastric surgery [7–9].

To the best of our knowledge, five English language publications have reported laparoscopically treated bronchogenic cyst located close to the EGJ (Table 1) [2, 10–13]. In two of these five cases, the surgeons used enucleation of the tumor with mucosa integrity, because mucosa was intact. In two other cases that did not directly involve the true EGJ, the tumors were resected using staplers. Only Kurokawa et al. [13] reported a manual full-thickness excision, but they closed the defect using a stapler, with no description of anti-reflux measures. In the present case, the tumor exhibited extraluminal growth with mucosal erosion; therefore, enucleation seemed inappropriate. The indication for laparoscopic and endoscopic cooperative surgery or percutaneous intragastric endoscopy is usually an intraluminal growing tumor. Finally, we chose full-thickness resection with hand-sewn closure. One crucial problem of this procedure may be exposure of the intragastric contents to the abdominal cavity. However, we believed that the probability of a malignant tumor was very low considering the imaging findings and therefore selected the herein described procedure. The technique with which we established and confirmed tightness during closure was very delicate. Intraoperative gastroendoscopy was helpful to calibrate the luminal caliber of the EGJ. Nevertheless, the lower esophageal sphincter function was partially lost, and we added partial fundoplication (Toupet fundoplication) to prevent postoperative reflux. The Thal-Hatafuku cardioplasty is an effective procedure for megaesophagus due to achalasia and was a plausible choice for this situation. However, this patient might receive bariatric surgery (sleeve gastrectomy or gastric bypass) in the future, which would be much more challenging after a Thal-Hatafuku procedure. We therefore selected the method described here.

**Table 1:** Publications that have reported laparoscopically treated bronchogenic cyst located close to the EGJ

Case	Author	Year	Age/sex	Tumor location	Tumor size (mm)	Surgical procedure	Anti-reflux measure	Complication
1	Melo et al. [11]	2005	39/F	E < G	50	Wedge resection with stapler	None	None
2	Diaz Nieto et al. [7]	2009	67/M	E > G	60	Enucleation with mucosal integrity	None	None
3	Fernandez et al. [12]	2011	33/M	Lesser sac near EGJ	43	Enucleation	None	Chronic chest wall pain
4	Ballehaninna UK et al. [2]	2013	40/F	E = G	50	Enucleation with mucosal integrity	Closure of muscular defect	None
5	Kurokawa et al. [13]	2013	71/M	E < G	30	Extirpation and closure with stapler	None	None
6	Our case	2016	32/F	E < G	60	Full-thickness resection and hand-sewn closure	Toupet fundoplication	None

*E > G* located mainly on esophageal side, *E = G* located on EGJ, *E < G* located mainly on gastric side

We considered the patient’s obesity in our treatment strategy. Had the tumor been definitely diagnosed as benign and located at the greater curvature site, we could have performed a sleeve gastrectomy. Currently, the patient’s weight is controlled only by diet, but if its control becomes poor, surgical treatment may be required. If her esophageal reflux is still a problem at that time, Roux-en-Y gastric bypass may be a first choice rather than sleeve gastrectomy.

## Conclusions

We have reported a case of a bronchogenic cyst arising from the EGJ treated with laparoscopic full-thickness resection with a unique suturing technique to prevent postoperative deformity and stenosis. When treating an SMT on the EGJ, the optimal surgical method should be chosen on an individual patient basis. This procedure may be an efficient option for such a tumor to maintain function or avoid excessive surgery.

## Consent

Written informed consent was obtained from the patient for publication of this case report and any accompanying images. A copy of the written consent is available for review by the Editor-in-Chief of this journal.

## Abbreviations

EGJ: esophagogastric junction
SMT: submucosal tumor
